# Estimated Breeding Values of Beef Sires Can Predict Performance of Beef-Cross-Dairy Progeny

**DOI:** 10.3389/fgene.2021.712715

**Published:** 2021-09-30

**Authors:** Natalia Martín, Lucy Coleman, Nicolás López-Villalobos, Nicola Schreurs, Stephen Morris, Hugh Blair, Julie McDade, Penny Back, Rebecca Hickson

**Affiliations:** ^1^School of Agriculture and Environment, Massey University, Palmerston North, New Zealand; ^2^Greenlea Premier Meats Ltd., Hamilton, New Zealand

**Keywords:** beef-on-dairy, carcass, crossbreeding, dairy-beef, genetic evaluation, gestation length, liveweight, progeny test

## Abstract

On average, half of the animal’s estimated breeding value (EBV) is passed on to their progeny. However, it is not known how the performance of beef-cross-dairy cattle relates to the EBV of their beef sire. Such information is required to determine the genetic potential of beef sires selected based on existing EBV to be used on dairy cows in New Zealand. This study evaluated the relationship between the EBV of 30 Angus and 34 Hereford sires and the performance of their progeny for birth, growth, and carcass traits, *via* progeny testing of 975 beef-cross-dairy offspring born to dairy cows and grown on hill country pasture. Overall, BREEDPLAN EBV did predict progeny performance of the beef-cross-dairy cattle from this study. Gestation length and birthweight increased with increasing sire EBV (mean 0.37–0.62days and 0.52–0.64kg, respectively, *p*<0.05). Age at weaning decreased with increasing sire EBV for liveweight at 200days (0.17–0.21days per extra kilo of sire EBV, *p*<0.05) but sire EBV for liveweight at 200days had no effect on the liveweight of the progeny at 200days for either breed (*p*>0.05). Liveweight increased with sire EBV for liveweight at 400, 600, and 800days, by a similar amount for both breeds (between 0.23 and 0.42kg increase in progeny liveweight per extra kilo of sire EBV, *p*<0.05). The relationships were more inconsistent for carcass traits. For Hereford, carcass weight and eye muscle area increased with increasing sire EBV (0.27kg and 0.70cm^2^, respectively, *p*<0.05). For Angus, marble score increased by 0.10 with 1% extra in sire EBV for intramuscular fat (*p*<0.05). Rib fat depth tended to increase with sire EBV for both breeds (*p*<0.1). EBV derived from beef-breed data work in dairy-beef systems but maybe slightly less than the expected 0.5units of performance per unit of EBV. New Zealand farmers should consider BREEDPLAN EBV when selecting sires to mate dairy cows or when buying beef-cross-dairy calves for beef production, to ensure the resulting calves are born safely and on time and then grow well to produce carcasses of suitable meat and fat composition.

## Introduction

Estimated breeding values (EBV) are predictions of the relative genetic merit of an animal for a particular trait (Bourdon, 2014). The EBV for most beef cattle breeds in New Zealand for a range of economically important traits are produced by BREEDPLAN (Agricultural Business Research Institute, University of New England, Armidale, Australia), and these are breed-specific, meaning that although the traits analyzed are common among breeds, the values are not directly comparable across breeds because they are analyzed separately and have different reference points. The EBV are calculated based on phenotypic records of the animal and relatives, and records are limited to those from registered, purebred cattle, which are almost exclusively in bull-breeding nucleus herds. On average, half of the animal’s EBV is passed on to the animal’s progeny (Bourdon, 2014), and so, the regression of purebred progeny performance on sire EBV is expected to have a 0.5 slope. In addition, the expected regression between EBV and progeny performance will have a greater coefficient of determination (R^2^) in traits with higher heritability.

The value of using EBV generated from data collected in purebred nucleus herds for selecting sires for use in a beef-on-dairy crossbreeding scheme is less clear. Genetically superior animals are expected to perform better than inferior counterparts, particularly for weight, fat, and carcass traits (Afolayan et al., 2007; McIntyre et al., 2009). However, the genetic correlation between purebred and crossbred performance is lower than unity for many traits, due to genotype-by-genotype interaction, genotype-by-environment interaction, and different definition of traits (Wientjes and Calus, 2017). Crossbred animals will show some degree of heterosis and complementarity (Bourdon, 2014), phenotypic differences may appear from one environment to another for the same genotype (Charteris et al., 1997), and the same trait may be measured differently between animals and systems (Wientjes and Calus, 2017). If this occurs, the relative ranking or scaling of breeds, sires within each breed and their crossbred progeny may change in different locations or farm systems (Charteris et al., 1997; Morris and Smeaton, 2009; Santana et al., 2013), and this should be reflected in the relationship between the EBV of the sire and the actual performance of the progeny.

There are important differences between the beef and dairy-beef systems in New Zealand, particularly around rearing, weaning, and finishing age. In a beef cow-calf system, calves are reared on their dams until weaning at a set date, around 5–7months of age [150–210days and 180–240kg liveweight (Geenty and Morris, 2017)]. In contrast in a dairy-beef system, calves are “artificially” reared on an allowance of 4–5L of milk per day until weaning at a set weight, usually of 80–100kg liveweight, achieved at 8–12weeks of age [56–84days (Muir et al., 2000; Geenty and Morris, 2017)]. By their first autumn (around 200days), cattle from dairy-beef systems are much lighter than cattle from cow-calf systems, and this carries through to finishing. Consequently, beef-bred cattle have a target slaughter age of 18–22months (Geenty and Morris, 2017), whereas beef-cross-dairy cattle are often slaughtered after their second winter to achieve target weights and avoid penalties associated with leanness and conformation (Bown et al., 2016).

Around 66% of the cattle slaughtered for beef production in New Zealand originate from dairy farms (Beef+Lamb New Zealand Economic Service, 2019; Ministry for Primary Industries, 2020; van Selm et al., 2021), and there is great industry interest to increase the proportion of surplus dairy calves that are reared for beef production, which will further raise this percentage. Thus, there is a strong need for genetic improvement of dairy-origin cattle in their potential for growth and carcass traits. To date, there is no information to quantify how useful the available EBV are to select beef bulls on their potential as terminal sires in a dairy-beef cattle system.

Previous studies in New Zealand (Martín et al., 2020, 2021; Coleman et al., 2021) have demonstrated considerable variation in performance among Angus and Hereford sires for birth, growth, and carcass traits. However, it was not explored how the sire performance related to their EBV. Given the considerable differences between beef and dairy-beef cattle systems mentioned above, it is possible that scaling or re-ranking of sires may occur. Such information is required in order to determine the genetic potential of beef sires selected based on existing EBV to be used on dairy cows, as these existing beef EBV may not be able to predict the performance of the crossbred progeny in a dairy-beef cattle system. Quantifying this relationship will assist with the development of future breeding programs for beef production in New Zealand, that include the use of beef-on-dairy crossbreeding systems.

The hypothesis of this study was that the performance of beef-cross-dairy offspring is predicted by their sire’s EBV. Therefore, this study aimed to evaluate the relationship between Angus and Hereford sires’ EBV and the performance of their progeny for birth traits (gestation length and birthweight), growth at different ages (liveweight at 200, 400, 600, and 800days of age), and carcass traits (carcass weight, eye muscle area, rib fat, and marble score), *via* progeny testing of beef-cross-dairy offspring born to dairy cows and grown on hill country pasture for beef production.

## Materials and Methodology

The animals reported here are a subset of animals for which growth and carcass traits were previously reported (Martín et al., 2020, 2021; Coleman et al., 2021). The animal study was conducted at Limestone Downs, near Port Waikato, New Zealand (37°28'S, 174°45'E). The study and all handling procedures were reviewed and approved by the Massey University Animal Ethics Committee (15/65 and 18/50). Animals were processed commercially through Greenlea Premier Meats Ltd., Hamilton plant, New Zealand (37°48'S 175°15'E), according to standard New Zealand industry practice (Animal and Animal Products Directorate, 2017).

### Animals and Management

#### Sires

The Angus and Hereford sires used in this experiment were selected from those nominated for progeny testing by cattle breeders in New Zealand. Sires were selected on the basis of their EBV prior to each mating. The EBV are generated by BREEDPLAN for Angus and Hereford bulls within breed, as part of the trans-Tasman Angus or Hereford genetic evaluations. The EBV for each sire were obtained from the online databases of the New Zealand Angus and Hereford breed associations^1^ and have been previously reported (Martín et al., 2020, 2021; Coleman et al., 2021). The data collected in this experiment were not included for the calculation of the BREEDPLAN EBV for these sires, and so, EBV values are independent of the progeny results obtained.

Within each breed, a spread of birthweight, gestation length, and liveweight at 600days of age (600d) EBV was achieved, except that birthweight EBV was restricted to the lightest 50% of the breed at the time of selection to prevent potential calving difficulty. When similar sires were available, those with superior EBV for intramuscular fat (IMF) and eye muscle area (EMA) were selected. There were a total of 31 Angus and 34 Hereford bulls used over 2 breeding seasons. Mean and range of EBV for birth, liveweight, carcass, and meat traits by breed of sire are presented in Table 1.

**Table 1:** Estimated breeding values (EBV; mean±SD), with total range, accuracy (mean and range), and percentile bands for gestation length, liveweight (at birth, 200, 400, and 600days of age), and carcass traits (carcass weight, eye muscle area, rib fat, and intramuscular fat), for 31 Angus and 34 Hereford sires.

Trait	Angus					Hereford				
	n	EBV	Range	Accuracy	Bands	n	EBV	Range	Accuracy	Bands
Gestation length (days)	31	−5.6±2.5	−10.1 to −0.4	89% (61–99)	1–95th	34	−1.5±3.4	−9.8 to 4.6	82% (45–99)	0–100th
Birthweight (kg)	31	2.6±1.5	−0.3 to 5.7	92% (71–99)	0–85th	34	2.2±1.8	−2.5 to 6.8	93% (74–99)	0–95th
200d weight (kg)	31	41±9	23 to 59	89% (70–99)	1–100th	34	30±8	18 to 48	91% (69–99)	0–99th
400d weight (kg)	31	78±13	56 to 110	89% (70–99)	1–99th	34	55±14	31 to 79	91% (69–99)	1–99th
600d weight (kg)	31	101±18	70 to 135	89% (70–99)	5–99th	34	74±20	35 to 114	91% (69–99)	0–100th
Carcass weight (kg)	31	52±15	26 to 80	81% (65–98)	1–99th	34	54±15	25 to 84	82% (58–98)	0–100th
Eye muscle area (cm^2^)	31	5.2±2.2	−0.3 to 9.7	80% (62–98)	1–100th	34	3.1±2.0	0.3 to 8.0	71% (50–96)	0–100th
Rib fat (mm)	31	0.9±1.9	−2.0 to 6.1	82% (64–98)	0–99th	34	0.8±1.0	−1.8 to 2.7	74% (52–97)	1–100th
Intramuscular fat (%)	31	1.5±1.4	−2.1 to 4.4	78% (56–98)	0−100th	34	0.4±0.7	−1.0 to 2.0	73% (38–97)	1–100th

*n*: number of sires used at mating; final number of sires included for data analysis: 30 Angus and 34 Hereford. Bands: fit of EBV within the percentile bands for 2018 born calves for each breed, where 0 is the top value and 100 is the bottom value for the trait. Updated in April 2020 from BREEDPLAN (Agricultural Business Research Institute, University of New England, Armidale, Australia; https://breedplan.une.edu.au/).

#### Dams

Lactating, mixed-aged cows (over 2years old and multiparous) were individually inseminated with semen from the selected sires. Cows were predominantly Holstein-Friesian or Holstein-Friesian-cross-Jersey crossbred. Semen was rotationally allocated to mating days and randomly allocated to cows in estrus on each mating day, to achieve random mating with semen from all sires distributed throughout the mating period. Cows were bred for 63days in 2015 and 54days in 2016.

#### Progeny

Angus-sired and Hereford-sired singleton calves born to mixed-aged dairy cows in spring 2016 (*n*=512) and 2017 (*n*=463) were included in the study. Mean birth date was August 6, 2016, (SD 18days) and August 8, 2017 (SD 16days). Calves born in the previous 24h were collected daily at approximately 10am and brought to the calf rearing shed.

In 2016, early-born calves (*n*=119) were sent to a commercial calf rearer (approximately 140km southeast of the farm, 37° 56'S 175° 39'E) at a minimum of 7days old. These calves were reared on an allowance of 3L of milk/head twice a day for the first 3weeks and then 4L of milk/head/day until weaning. Calves were fed colostrum or whole milk for 6weeks and a 50:50 mixture of stored colostrum and milk powder (Ancalf, NZAgBiz, Hamilton, New Zealand) for the remainder of the time until weaning. All calves were offered *ad libitum* meal [16% Crude Protein (CP)] and had no access to pasture during the pre-weaning period. These calves were weighed weekly and weaned at a minimum of 75kg liveweight, before returning to the farm of birth at around 100kg.

The remainder of the calves born in 2016, and all calves born in 2017, were reared on the dairy farm. These calves were reared on an allowance of 4 liters of milk/head/day of whole milk, and calf meal (17–20% CP) was offered *ad libitum* during the transition from milk to pasture. Calves were weighed every 1–3weeks and weaned at a minimum of 85kg liveweight.

The resulting mean weaning liveweight of all calves in this study was 93.0kg (SD 7.0). Once weaned, calves were moved from the dairy farm to the adjacent sheep and beef hill country farm. Male calves were castrated before 4months of age.

At 4months of age (December of 2016 and 2017), at a mean age of 128.7days (SD 16.8), and a mean liveweight of 123.3kg (SD 15.5), calves were allocated into 6 grazing herds based on liveweight (light, intermediate, and heavy) and sex (heifer and steer) and balanced for sire so that, where possible, all sires were represented in each grazing herd within a year. In total, there were 12 grazing herds (2years×2 sexes×3 liveweight groups) and animals remained in those herds throughout the experiment until slaughter. All cattle were grazed on summer-dry hill country pasture under commercial conditions (Martín et al., 2020). Any animals that died or were removed from their contemporary group due to illness or escaping were removed from the experiment at that time, but previous measurements were included in the study.

Each grazing herd was slaughtered as a complete group on the same day, when the mean liveweight reached the slaughter target liveweight of 500kg for heifers and 600kg for steers (Martín et al., 2021). Heifers were slaughtered at a mean age of 819days of age (range 693–923, 27months old) and 519kg (SD 37) liveweight, while steers were slaughtered at a mean age of 885days of age (range 821–954, 29months old) and 613kg (SD 43) liveweight.

### Measurements

#### Birth Traits

Birth traits were recorded for all calves, whether alive or dead at the time of collection. Parentage was assigned using DNA parentage assignment (Zoetis, Dunedin, New Zealand). Gestation length (days) was calculated as the difference between insemination date and birth date. Insemination date was the date on which the DNA-assigned dam was inseminated using semen from the DNA-assigned sire. Date of birth was recorded as the date which the calf was brought into the calf rearing shed.

Birthweight was recorded on arrival to the calf rearing shed, prior to being fed, using a weigh crate (Prattley Industries Ltd., Temuka, New Zealand; weight scales model EziWeigh7i, Tru-Test, Auckland, New Zealand; load bars MP600, Tru-Test, Auckland, New Zealand).

#### Growth Traits

Prior to weaning, calves were weighed every 1–3weeks as they approached weaning weight, and on each occasion, calves over 85kg were weaned (75kg at the commercial rearer). Date and liveweight at weaning were recorded for each calf. Age at weaning was calculated as the difference between birth and weaning dates.

After weaning, calves were weighed on the farm using a weigh crate at a minimum of 2-monthly intervals, as described in Martín et al. (2020). Short-term fluctuations in liveweight were smoothed out by calculating centered moving averages of three liveweight records per animal using the Expand procedure (SAS 9.4, SAS Institute Inc., Cary, NC, United States). Predicted liveweights for each animal at 200d, 400d, 600d, and 800d were calculated by interpolation of the smoothed liveweight curves.

#### Carcass Traits

After slaughter, the bodies were dressed to New Zealand commercial specifications (Animal and Animal Products Directorate, 2017), and hot carcass weight (kg) was recorded prior to the carcasses going into the chiller (Martín et al., 2021). Carcasses were chilled (4±1°C) overnight, and the following morning, one side of the carcass was cut between the 12th and 13th rib to expose the eye muscle (*M. longissimus thoracis*) for in-chiller assessment of rib fat thickness (mm), eye muscle area (EMA in cm^2^), and marbling score. Marbling was scored on a scale from 0 (nil) to 9 (abundant) according to the AUS-MEAT/MSA reference standards (AUS-MEAT Limited, 2018).

### Statistical Analysis

#### Data Cleaning

Sires with a minimum of five progeny records were included in the analysis for that trait, resulting in 1 Angus sire excluded for all traits, 1 Angus sire excluded for all traits except birthweight, and 1 Hereford sire excluded for marble score.

#### Progeny Mean Calculations

Linear mixed models were used to estimate least-squares means for progeny groups using the statistical package SAS 9.4 (SAS Institute Inc. 2013, Cary, NC, United States).

The models for birth traits (gestation length and birthweight) included the fixed effect of sire within breed and the random effect of contemporary group. Contemporary group (*n*=8, with 101 to 149 animals in each group) was defined as the group of animals born in the same year (*n*=2, 2016 and 2017), of the same sex (*n*=2, heifer and steer) that were progeny of sires of the same breed (*n*=2, Angus and Hereford).

The model for weaning age included the fixed effect of sire within breed and the random effect of contemporary group. Contemporary group (*n*=12, with 25 to 117 animals in each group) was defined as the group of animals born in the same year (*n*=2, 2016 and 2017), of the same sex (*n*=2, heifer and steer) that were progeny of sires of the same breed (*n*=2, Angus and Hereford) and were reared at the same location (*n*=2, commercial rearer and dairy farm). Weaning weight was fitted as a covariate for weaning age.

The models for liveweight (at 200d, 400d, 600d, and 800d) included the fixed effect of sire within breed and the random effect of contemporary group. Contemporary group (*n*=24, with 25 to 47 animals in each group) was defined as the group of animals grazing in the same herd (*n*=12, 2years×2 sexes×3 liveweight groups), that were progeny of sires of the same breed (*n*=2, Angus and Hereford).

The models for carcass traits (carcass weight, EMA, rib fat depth and marble scores) included the fixed effect of sire within breed and the random effect of contemporary group. Contemporary group (*n*=24, with 24 to 41 animals in each group) was the same as that for liveweight. Age deviation at slaughter (within contemporary group) was fitted as a covariate for carcass weight. Carcass weight was fitted as a covariate for EMA, rib fat depth, and marble score.

#### Regressions of Progeny Means on Sire EBV

Least-squares means of beef-cross-dairy progeny groups for birth, growth, and carcass traits were regressed against sire EBV using linear regressions, to test the slope of the regression to be greater than 0. The regressions with 95% confidence intervals were done separately for each breed of sire and were weighted by the number of progeny of each sire for each trait. Sire EBV for liveweight at 200d was used to predict least-squares means of the progeny groups for weaning age. Sire EBV for liveweight at 600d was used to predict least-squares means of the progeny groups for liveweight at 800d. Sire EBV for IMF was used to predict least-squares means of the progeny groups for marble score.

## Results

The total number of sires and progeny, and mean and range of the least-square means of beef-cross-dairy progeny groups for birth, liveweight, and carcass traits is presented in Table 2.

**Table 2:** Number of sires and progeny, mean (± SD), and range of the least-squares means of beef-cross-dairy progeny groups for birth (gestation length and birthweight), growth (age at weaning and liveweight (LWT) at 200, 400, 600, and 800days of age), and carcass traits (carcass weight, eye muscle area (EMA), rib fat depth, and marble score), for 30 Angus and 34 Hereford sires.

Progeny mean	Angus				Hereford			
	*n* sires	*n* progeny	Mean±SD	Range	*n* sires	*n* progeny	Mean±SD	Range
Gestation length (days)	29	405	279.8±1.5	276.4 to 282.9	34	455	282.9±2.8	276.1 to 289.0
LWT at birth (kg)	30	463	36.2±1.6	33.6 to 40.2	34	512	37.3±1.9	33.7 to 43.1
Age at weaning (days)	29	407	82.2±3.3	76.8 to 88.0	34	458	80.9±4.3	70.2 to 88.2
LWT at 200d (kg)	29	406	158.4±5.3	148.4 to 168.5	34	460	158.7±5.1	150.1 to 172.3
LWT at 400d (kg)	29	394	284.1±7.9	269.5 to 299.3	34	457	283.4±8.8	264.1 to 298.3
LWT at 600d (kg)	29	379	425.2±13.6	401.9 to 465.1	34	446	426.0±12.0	402.5 to 447.8
LWT at 800d (kg)	29	329	502.5±14.2	475.7 to 540.7	34	397	505.4±15.8	470.4 to 544.1
Carcass weight (kg)	29	369	276.4±9.2	258.5 to 304.8	34	429	277.0±9.3	260.7 to 295.6
EMA (cm^2^)	29	367	73.8±3.0	66.8 to 79.0	34	428	73.2±3.7	66.9 to 82.9
Rib fat depth (mm)	29	366	7.2±1.2	4.8 to 9.2	34	428	7.7±1.5	4.9 to 11.3
Marble score	29	331	1.1±0.3	0.5 to 1.6	33	384	0.8±0.3	0.2 to 1.5

### Regression of Progeny Means on Sire EBV for Birth Traits

**Figure 1:**
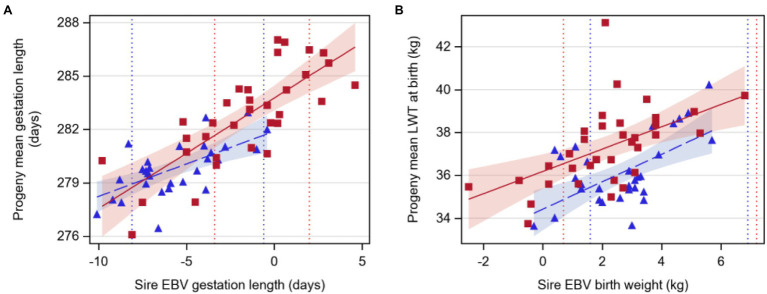
Regression of least-squares means of beef-cross-dairy progeny groups on sire EBV for: **(A)** gestation length (days) and **(B)** liveweight at birth (LWT, kilograms). Individual sires are represented by one data point (Angus, 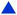 triangles, *n*=30; Hereford, 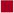 red squares, *n*=34), and the regression lines by breed are indicated by lines (Angus, 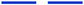 dashed line; Hereford, 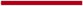 solid line). Shaded areas indicate 95% confidence intervals for the expected regression line for each breed (Angus, 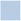 blue; Hereford, 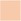 red). Colored dotted lines indicate 5 and 95th EBV percentiles for 2018 born calves within the BREEDPLAN population for each breed (Angus, 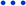 blue; Hereford, 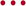 red). Details of regression coefficients are presented in Table 3.

Gestation length increased by 0.37 or 0.62days for a 1-day increase in sire EBV for Angus and Hereford sires, respectively (*p*<0.05, Table 3; Figure 1). Birthweight increased by 0.64 or 0.52kg per 1kg extra in sire EBV for Angus and Hereford sires, respectively (*p*<0.05).

**Table 3:** Estimates of regression coefficients (intercept and slope) of least-squares means of beef-cross-dairy progeny groups for birth traits (gestation length and birthweight) on corresponding sire EBV, for 30 Angus and 34 Hereford sires.

Progeny mean	Angus				Hereford			
	Intercept	Slope	*p*-value	R^2^	Intercept	Slope	*p*-value	R^2^
Gestation length (days)	281.9±0.5	0.37±0.09	<0.001	0.40	283.8±0.3	0.62±0.10	<0.001	0.57
Birthweight (kg)	34.4±0.5	0.64±0.15	<0.001	0.39	36.2±0.4	0.52±0.14	<0.001	0.32

R^2^: coefficient of determination.

### Regression of Progeny Means on Sire EBV for Growth Traits

**Figure 2:**
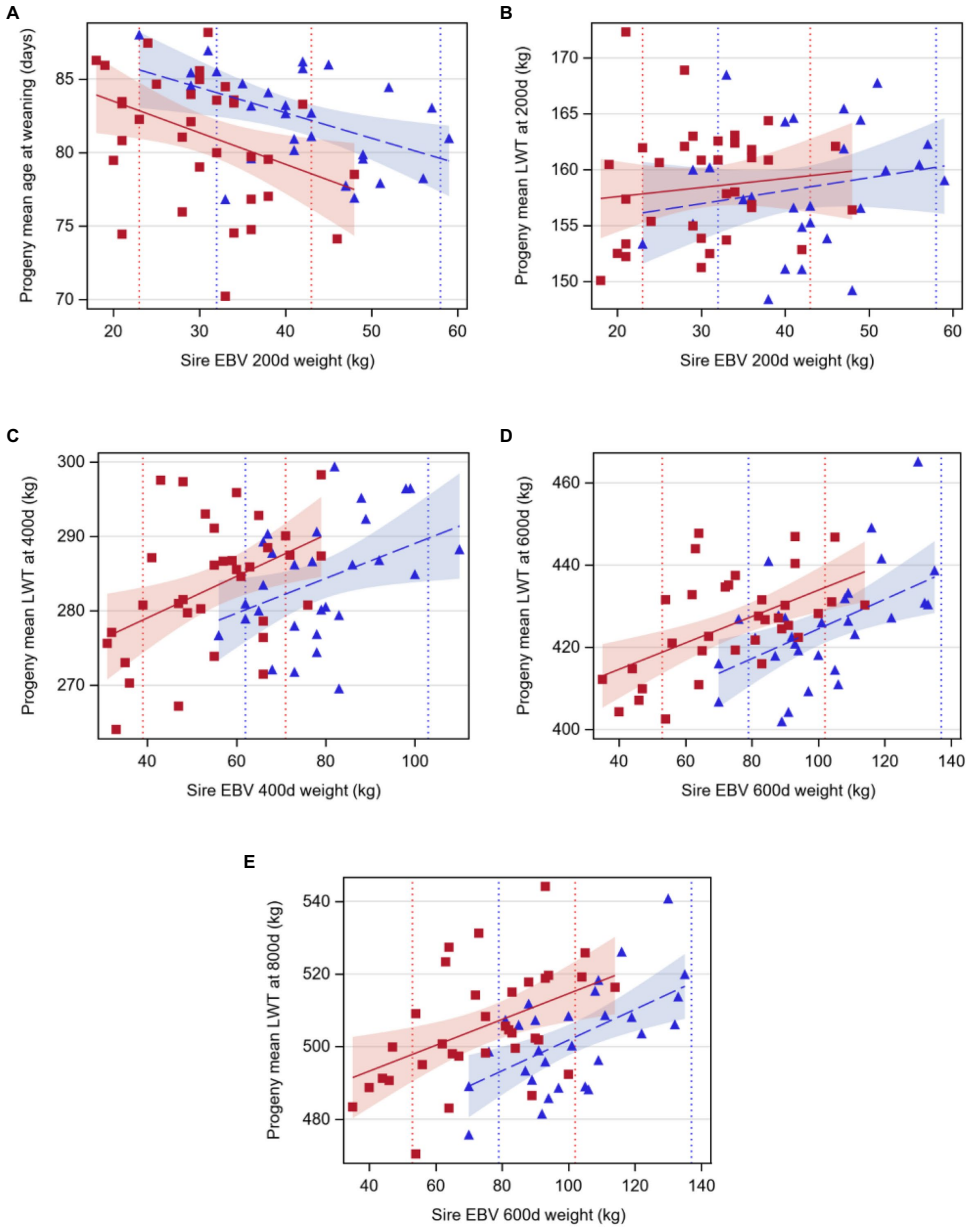
Regression of least-squares means of beef-cross-dairy progeny groups on sire EBV for: **(A)** age at weaning (days) on weight at 200days (kilograms), **(B)** liveweight at 200days (LWT, kilograms), **(C)** liveweight at 400days (LWT, kilograms), **(D)** liveweight at 600days (LWT, kilograms), and **(E)** liveweight at 800days on weight at 600days (LWT, kilograms). Individual sires are represented by one data point (Angus, 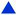 blue triangles, *n*=29; Hereford, 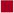 red squares, *n*=34), and the regression lines by breed are indicated by lines (Angus, 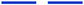 dashed line; Hereford, 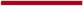 solid line). Shaded areas indicate 95% confidence intervals for the expected regression line for each breed (Angus, 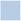 blue; Hereford, 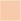 red). Colored dotted lines indicate 5 and 95th EBV percentiles for 2018 born calves within the BREEDPLAN population for each breed (Angus, 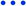 blue; Hereford, 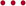 red). Details of regression coefficients are presented in Table 4.

Age at weaning decreased by 0.17 or 0.21days per 1kg extra in sire EBV for liveweight at 200d for Angus and Hereford, respectively (*p*<0.05, Table 4; Figure 2). However, sire EBV for liveweight at 200d had no effect on the liveweight of the progeny at 200d for either breed (*p*>0.05). Liveweight increased with EBV at 400d, 600d, and 800d, by a similar amount for both breeds (*p*<0.05).

**Table 4:** Estimates of regression coefficients (intercept and slope) of least-squares means of beef-cross-dairy progeny groups for growth traits (age at weaning and liveweight at 200, 400, 600, and 800days of age) on corresponding sire EBV for liveweight, for 29 Angus and 34 Hereford sires.

Progeny mean	Angus				Hereford			
	Intercept	Slope	*p*-value	R^2^	Intercept	Slope	*p*-value	R^2^
Age at weaning (days)^‡^	89.6±2.6	−0.17±0.06	0.009	0.23	87.8±2.7	−0.21±0.08	0.015	0.17
LWT at 200d (kg)	153.5±4.6	0.12±0.11	0.291	0.04	156.0±3.7	0.08±0.11	0.482	0.02
LWT at 400d (kg)	265.6±8.1	0.23±0.10	0.029	0.16	267.9±5.7	0.28±0.10	0.008	0.20
LWT at 600d (kg)	388.4±11.5	0.36±0.11	0.003	0.28	401.8±6.6	0.32±0.08	<0.001	0.32
LWT at 800d (kg)^¥^	459.5±11.9	0.42±0.11	0.001	0.33	479.0±9.5	0.36±0.12	0.006	0.21

R^2^: coefficient of determination.

‡ Progeny mean for age at weaning was regressed with sire EBV for weight at 200d.

¥ Progeny mean for LWT at 800d was regressed with sire EBV for weight at 600d.

### Regression of Progeny Means on Sire EBV for Carcass Traits

**Figure 3:**
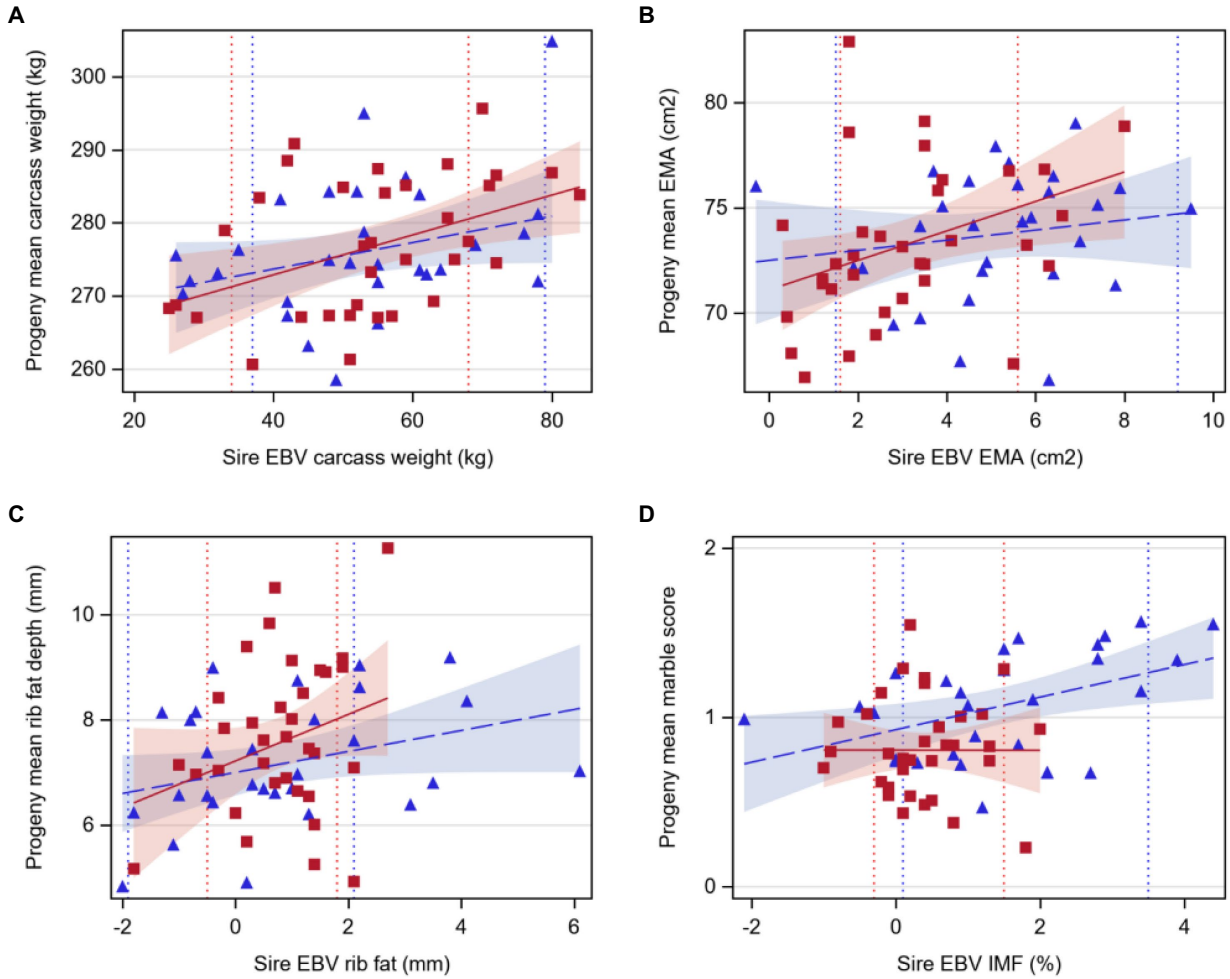
Regression of least-squares means of beef-cross-dairy progeny groups on sire EBV for: **(A)** carcass weight (kg), **(B)** eye muscle area (EMA, cm^2^), **(C)** rib fat depth (mm), and **(D)** marble score (on intramuscular fat, IMF %). Individual sires are represented by one data point (Angus, 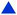 blue triangles, *n*=29; Hereford, 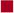 red squares, *n*=34), and the regression lines by breed are indicated by lines (Angus, 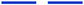 dashed line; Hereford, 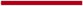 solid line). Shaded areas indicate 95% confidence intervals for the expected regression line for each breed (Angus, 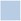 blue; Hereford, 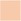 red). Colored dotted lines indicate 5 and 95th EBV percentiles for 2018 born calves within the BREEDPLAN population for each breed (Angus, 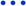 blue; Hereford, 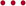 red). Details of regression coefficients are presented in Table 5.

For Hereford, carcass weight increased by 0.27kg per 1kg extra in sire EBV (*p*<0.05, Table 5; Figure 3), EMA increased by 0.70cm^2^ with 1cm^2^ extra in sire EBV (*p*<0.05), and rib fat depth tended to increase with sire EBV (*p*<0.1), but there was no relationship between marble score and sire EBV for IMF (*p*>0.05). For Angus, marble score increased by 0.10 with 1% extra in sire EBV for IMF (*p*<0.05, Table 5; Figure 3), while carcass weight and rib fat depth tended to increase with sire EBV (*p*<0.1), and there was no relationship between EMA and sire EBV (*p*>0.05).

**Table 5:** Estimates of regression coefficients (intercept and slope) of least-squares means of beef-cross-dairy progeny groups for carcass traits (carcass weight, eye muscle area (EMA), rib fat depth, and marble score) on corresponding sire EBV, for 29 Angus and 34 Hereford sires.

Progeny mean	Angus				Hereford			
	Intercept	Slope	*p*-value	R^2^	Intercept	Slope	*p*-value	R^2^
Carcass weight (kg)	266.4±5.4	0.18±0.10	0.079	0.11	262.0±5.6	0.27±0.10	0.008	0.20
EMA (cm^2^)	72.5±1.4	0.24±0.26	0.356	0.03	71.1±1.1	0.70±0.30	0.025	0.15
Rib fat depth (mm)	7.0±0.2	0.20±0.11	0.070	0.12	7.2±0.3	0.44±0.25	0.091	0.09
Marble score^‡^	0.93±0.07	0.10±0.04	0.012	0.21	0.81±0.06	0±0.07	0.993	0.00

R^2^: coefficient of determination.

‡ Progeny mean for marble score was regressed with sire EBV for intramuscular fat.

## Discussion

Sire EBV had a positive association with progeny gestation length and birthweight for both breeds, indicating that choosing sires with lower EBV will reduce the gestation length and birthweight of calves. For gestation length, the association was stronger for the Hereford sires than the Angus sires (R^2^=57 and 40%, respectively), because there was a greater spread and more sires in the top 5% of the breed. The good match between EBV and actual performance indicates that these are likely to be the same trait being assessed in both purebred Angus or Hereford cattle and beef-cross-dairy cattle. Gestation length records are readily obtained in dairy herds through the widespread use of artificial breeding in New Zealand [over 70% of dairy cows (LIC and DairyNZ, 2020)], and this information could be used to provide information on the sires to assist in evaluating genetic merit for gestation length.

For birthweight, the restriction during selection to the lighter half of each breed meant that the sires included in this study were biased toward the lighter end of the breed, and consequently, the associations of progeny and sire EBV were low-to-moderate for both breeds (R^2^=32–39%). Regardless, the associations were positive and close to the expected 0.5 slope, and so, BREEDPLAN EBV can be used to manage birthweight when selecting beef sires for use in the dairy industry. A similar approach has been used in other studies to avoid calving difficulties (Berry et al., 2019; Eriksson et al., 2020).

The earlier weaning age for sires with greater EBV for liveweight at 200d is the result of faster pre-weaning growth of these calves. It should be noted that birthweight was not related to 200d weight EBV (*p*>0.05, data not shown), so the earlier weaning age was not simply a birthweight effect. Weaning age can have important implications at the dairy farm, where there is an explicit cost involved in milk feeding. The findings also align with the industry recommendations for calf rearing in New Zealand, based predominantly on Holstein-Friesian bull calves, where calves that grow faster are typically weaned earlier (Muir et al., 2001, 2002).

The lack of association between sire EBV and progeny liveweight at 200d was foreseeable given the differences in breeds (beef vs. beef-cross-dairy) and rearing systems used to calculate sire EBV and progeny liveweight. Angus and Hereford sires’ EBV for liveweight at 200d are derived from weaning weight records on purebred beef calves, reared in cow-calf systems where calves are reared on their mother until 200d (Geenty and Morris, 2017). This EBV measures pre-weaning growth, which is influenced by the calf’s growth potential and feeding intensity, and the dam’s milk production (Asheim et al., 2016; Geenty and Morris, 2017). Recent results from a Beef Progeny Test in New Zealand, where 984 progeny of 52 sires of Angus, Hereford, Stabilizer, Simmental, and Charolais breeds were considered for growth and carcass traits, showed that 1kg extra in the sire EBV for 200d weight could predict 0.49kg increase in liveweight of the calves at weaning at 200d (Beef+Lamb New Zealand Genetics, 2019). In that study, mean liveweight at weaning was 206.1kg. In comparison, calves from the present experiment were removed from their dams within 24h of birth, and group fed on an allowance of 4–6L of milk/head/day until weaning. Weaning occurred at a fixed liveweight, rather than a fixed age as it happens in cow-calf rearing systems. This meant that lighter or slower-growing calves got fed milk for more days to achieve the target weaning weight and so may have had an advantage compared with faster-growing calves, effectively creating a genotype-by-environment interaction that favored the slower-growing calves. The unequal treatment of calves perhaps prevented a good assessment of the growth potential at 200d weight of the calves, but it reflects the reality of the dairy-beef systems and highlights the importance of including weaning age in the assessed traits.

After weaning, the calves in this study went on a pasture-based diet, which was of low quality over the summer [metabolizable energy (ME) of 9.2MJ per kilogram of dry matter (kgDM), CP 12.6 %DM (Martín et al., 2020)]. Low-quality pasture likely restricted animal performance, because calves grew at an average of 0.55kg/d from weaning at 81.5–200d, compared with 0.69kg/d pre-weaning (0–81.5d). Young cattle require pasture with greater than 11.4 MJME/kgDM and covers higher than 2,200 kgDM per hectare to grow at the target rate of 1kg/d (Geenty and Morris, 2017) and to express their genetic potential for growth, or alternatively when pasture quality is poor, they require supplementary concentrate feed to increase liveweight gain (Pettigrew et al., 2017). Therefore, there was no maternal influence through milk production on calf growth, and liveweight at 200d was the result of post-weaning liveweight gain restricted by the poor-quality diet. Additionally, the prolonged artificial rearing of the slower-growing calves in this dairy-beef system enabled them to achieve similar liveweights to faster-growing calves up to 200d. Consequently, 200d liveweight EBV is a useful predictor of weaning age in a dairy-beef system, but is not a good indicator of liveweight at 200d.

By 400d, liveweight of calves was again reflective of their sire’s EBV, although the regression coefficient was less than the 0.41 and 0.45 reported for 400d and 600d weight in the New Zealand Beef Progeny Test mentioned earlier (Beef+Lamb New Zealand Genetics, 2019). When comparing the beef-cross-dairy progeny liveweights at 800d with sire EBV for liveweight at 600d, the association was much stronger. The increased regression coefficient with increasing age likely reflected the decreasing influence of the longer milk-feeding period of slow-growing calves in early life. Additionally, the cattle were closer to mature size by 800d, having grown relatively slow during their life (0.43kg/d from 200d to 800d). The low-quality pasture would have restricted animal performance (Burke et al., 2002) and likely resulted in a smaller increase in progeny performance with increasing sire EBV than may have been achieved in an environment that provided greater quantity and quality of feed, without necessarily changing the ranking of the sires (Hammami et al., 2009; McIntyre et al., 2009).

Increasing sire EBV for carcass weight and EMA was associated with increases in the respective traits in the progeny of the Hereford sires in this experiment, but this was not the case for the Angus sires. For both breeds, sire EBV had a positive trend on rib fat depth. The R^2^ value was low for both breeds, but this result was generally consistent with the result from the Beef Progeny Test previously mentioned (Beef+Lamb New Zealand Genetics, 2019). One possible explanation for the better predictive ability of liveweight compared with any carcass EBV is that there is much more phenotypic information available to predict the EBV for liveweight, and the data to derive these EBV have been recorded for a longer period of time (Cundiff et al., 2007). This was reflected in the lower accuracy of the carcass vs. liveweight EBV of the sires used: Accuracy for liveweight EBV ranged from 69 to 99% (mean 89% for Angus and 91% for Hereford sires), while accuracies for carcass weight EBV ranged from 58 to 98% (mean 81% for Angus and 82% for Hereford sires) and for EMA EBV from 50 to 98% (mean 80% for Angus and 71% for Hereford sires). Further, sire EBV for EMA, as well as rib fat depth and IMF, are based on ultrasound measurements on live animals at 18months of age as well as measurements on the carcass for related animals processed in Australia (Johnston et al., 1999; BREEDPLAN, 2015), whereas the progeny group means presented here were based only on measurements collected at processing at around 28months of age. Even though the genetic correlation of ultrasound and carcass measures is moderate to strong (Reverter et al., 2000), there is considerable variation in the values of the correlations [e.g., for Angus and Hereford breeds, rib fat depth r_g_=0.02 to 0.99, EMA r_g_=0.16 to 0.94, IMF r_g_=0.28 to 0.93 (Reverter et al., 2000; Su et al., 2017)]. This will inevitably affect the accuracy of the EBV (agreement between true and predicted values) and highlights the need to improve the information gathered to generate the EBV for carcass traits. Yet, precision (correct ranking of animals) is more important than accuracy for genetic evaluations (Herring and Kemp, 2001). Therefore, differences in the ability of the EBV to predict progeny performance for carcass traits were somewhat expected.

Sire EBV for IMF had a low-to-moderate positive association with marble score for the Angus sires in this experiment (slope=0.10; R^2^=21%), but not for the Hereford sires. Angus sires used in the experiment had a wider range of EBV for IMF compared with Hereford sires (6.5 and 3.0% IMF spread of EBV, respectively, although this is a reflection of the spread within each breed rather than a difference in sampling). Moreover, Angus-sired progeny had higher marble scores compared with Hereford-sired cattle [0.21 scores greater, *p*<0.05 (Martín et al., 2021)], even though the range of scores was 0–3 for both breeds. Accordingly, more Angus sires had higher marble scores than Hereford sires in this experiment. This result may be explained by the physiological age at slaughter and the breeders’ emphasis on marbling. Even though both Angus and Hereford are considered early maturing breeds (Purchas, 2003; Bartoň et al., 2006), Angus cattle are known to reach physiological maturity at an earlier age (Chambaz et al., 2001) and to exhibit higher marbling than other beef breeds (Thonney, 2015). Given that the cattle in this study were slaughtered at a common age (which depended on the contemporary group reaching a mean target weight, rather than selecting those animals with the right finishing attributes, such as good fat cover over the back and tail), then it is possible that Hereford cattle were not as physiologically mature as the Angus cattle. This is supported by the fact that Hereford-sired cattle had longer carcasses than Angus-sired animals in this study [2.0cm difference, *p*<0.05 (Martín et al., 2021)], indicating that Hereford were larger-framed and less mature compared with Angus cattle, at the same carcass weight (Thonney, 2015). In addition, New Zealand Angus breeders have been putting emphasis on marbling through the AngusPure Index. To qualify for AngusPure grading premiums,^2^ heifers and steers require a minimum marbling score of 2. Even though cattle that are half dairy cannot qualify for this premium, greater marble scores can be achieved in beef-cross-dairy animals by selecting sires that rank highly in this breeding index.

One limitation of this experiment was that the maternal breed was not accounted for because there was scarce information on the dams. Dams were predominantly Holstein-Friesian or Holstein-Friesian-cross-Jersey crossbred. A greater proportion of Holstein-Friesian would produce heavier calves with faster growth rates and heavier carcasses, while a greater proportion of Jersey would produce smaller animals with slower growth rates, lighter carcasses, and higher marbling (Barton et al., 1994; Buchanan and Lenstra, 2015; Bown et al., 2016; Handcock et al., 2018). Nevertheless, it is unlikely that there would be a bias in the data from this study because sires were randomly allocated to cows, and analysis by Coleman et al. (2019) showed that the cows in this experiment had similar liveweights and milk production regardless of the sire they were bred to.

A further potential limitation is that sires used in this experiment were not a random sample, as they were selected for the project based on their EBV so that, within each breed, a spread of birthweight (restricted to the lighter 50%), gestation length, and liveweight at 600d was achieved. With greater selection intensity (smaller proportion of bulls selected from the total available), then the expected correlation between EBV in different systems decreases (Charteris, 1995). However, the selection to achieve a spread of EBV creates a more representative population than selection for the best EBV, and the EBV of the sires of both breeds did cover most of the range of possible EBV for each growth and carcass trait [spanning at least the percentiles 1–99th for most, except for Angus gestation length (1–95th), birthweight (0–85^th^), and liveweight at 600d (5–99th), and Hereford birthweight (0–95th)].

Lastly, there was variation in the EBV accuracy, which ranged between 45 and 99% for birth traits, 69–99% for growth traits, 50–98% for carcass size traits, and 38–98% for carcass fat traits. The traits examined also vary in their published heritability estimates, namely, 0.44–0.68 for gestation length, 0.25–0.45 for birthweight, 0.12–0.70 for liveweight, 0.23–0.54 for carcass weight, 0.20–0.47 for EMA, 0.25–0.45 for rib fat depth, 0.15–0.49 for IMF, and 0.17–0.48 for marbling (Angus New Zealand, n.d.; Baker et al., 1975, 1990; Van Vleck et al., 1992; Gregory et al., 1995; Reverter et al., 2000, 2003a,b; Winkelman and Spelman, 2001; Rios Utrera and Van Vleck, 2004; Afolayan et al., 2007; Warner et al., 2010; Amer et al., 2016; Su et al., 2017). Lower accuracy of the EBV, in addition to each sire having a low number of progeny (5–25 progeny for most traits), implies that both the available BREEDPLAN EBV and the measured phenotypic results could be an over- or under-representation of the real sire merit. Errors in the independent variable, in this case EBV, can cause significant bias in estimated regression coefficient (Robinson, 2005), reducing the regression slope. On the other hand, increasing progeny numbers evaluated per sire can increase the accuracy and correlation between sire EBV in different environments (Blair and Garrick, 1994). For example, assuming the genetic correlation of a trait across environments is 1.0 and a heritability of the trait of 0.25, increasing the number of progeny tested from 20 to 50 would increase the correlation between EBV for different environments from 0.38 to 0.76 (Charteris, 1995). Nevertheless, the number of progeny used in this study is comparable with other progeny tests done in New Zealand (Baker et al., 1975; Beef+Lamb New Zealand Genetics, 2019), and the large number of sires used allows for mendelian sampling effects on individual progeny group means.

## Conclusion

Overall, BREEDPLAN EBV did predict progeny performance of the beef-cross-dairy cattle from this study, indicating that EBV derived from beef-breed data work in dairy-beef systems but may result in slightly less than the expected 0.5 unit increase in performance per unit of EBV. This was particularly true for birth and growth traits, except for 200d liveweight for both Angus and Hereford breeds. The relationships were less consistent for carcass traits. Hereford sires had better associations with carcass size traits, while Angus sires had better associations with carcass fat traits. Although EBV for carcass traits are currently useful, improvement in the information used to generate them is still required to increase their accuracy and ability to predict progeny performance. Beef-cross-dairy cattle could also be included in joint genetic evaluations, given the strong dairy component in the cattle slaughtered in New Zealand. New Zealand farmers should consider BREEDPLAN EBV when selecting sires to mate to dairy cows or when buying beef-cross-dairy calves for beef production, to ensure the resulting calves are born safely and on time, and then grow well to produce carcasses of suitable meat and fat composition.

## Data Availability Statement

The raw data supporting the conclusions of this article will be made available on request to the corresponding author.

## Ethics Statement

The animal study was reviewed and approved by Massey University Animal Ethics Committee (Palmerston North, New Zealand). Written informed consent was obtained from the owners for the participation of their animals in this study.

## Author Contributions

RH, PB, NL-V, LC, and NM: conceptualization. NM, LC, RH, NL-V, PB, HB, NS, SM, and JM: methodology and writing – review and editing. NM and LC: investigation, data curation, and software. NM, LC, RH, and NL-V: formal analysis. NM and RH: validation, NM: visualization and writing – original draft preparation. RH, PB, NL-V, NS, SM, HB, and JM: supervision. NM, LC, RH, PB, and JM: project administration. RH, PB, and JM: resources. RH and JM: funding acquisition. All authors contributed to the article and approved the submitted version.

## Funding

This research was funded by Beef and Lamb New Zealand Genetics (DBPT2015). The primary author was funded by a R&D Fellowship Grant between Callaghan Innovation and Greenlea Premier Meats Limited-Massey University (GPMES1601/PROP-50347-FELLOW-GPMES) and the second author was funded by a Massey University Doctoral Scholarship. The contributions of the C. Alma Baker Trust NZ Ltd., Angus NZ, and NZ Hereford Association are gratefully acknowledged. The funders approved the design of the study but had no role in the collection, analyses or interpretation of data, in the writing of the manuscript, or in the decision to publish the results.

## Conflict of Interest

JM is employed by Greenlea Premier Meats Ltd. (Hamilton, New Zealand).

The remaining authors declare that the research was conducted in the absence of any commercial or financial relationships that could be construed as a potential conflict of interest.

## Publisher’s Note

All claims expressed in this article are solely those of the authors and do not necessarily represent those of their affiliated organizations, or those of the publisher, the editors and the reviewers. Any product that may be evaluated in this article, or claim that may be made by its manufacturer, is not guaranteed or endorsed by the publisher.

## Footnotes

^1^ https://breedplan.une.edu.au/

^2^ https://www.anguspure.co.nz/
